# Dietary Sodium Butyrate Improves Intestinal Health of Triploid *Oncorhynchus mykiss* Fed a Low Fish Meal Diet

**DOI:** 10.3390/biology12020145

**Published:** 2023-01-17

**Authors:** Siyuan Liu, Shuze Zhang, Yaling Wang, Shaoxia Lu, Shicheng Han, Yang Liu, Haibo Jiang, Chang’an Wang, Hongbai Liu

**Affiliations:** 1 Key Laboratory of Aquatic Animal Diseases and Immune Technology of Heilongjiang Province, Heilongjiang River Fisheries Research Institute, Chinese Academy of Fishery Sciences, Harbin 150070, China; 2 College of Fisheries and Life Science, Dalian Ocean University, Dalian 116023, China; 3 College of Animal Science and Technology, Northeast Agricultural University, Harbin 150006, China; 4 College of Fisheries and Life Science, Shanghai Ocean University, Shanghai 201306, China; 5 College of Animal Science, Guizhou University, Guiyang 550025, China

**Keywords:** sodium butyrate, triploid *Oncorhynchus mykiss*, intestinal health, low fish meal diet, intestinal immunity

## Abstract

**Simple Summary:**

Soybean meal is currently one of the most widely used ingredients in aquafeed due to its consistent supply, reasonable price, and well-balanced amino acid profile. However, anti-nutritional factors such as antigen proteins and soybean agglutinin in soybean meal can also cause intestinal barrier injury and inflammatory reactions in fish, harming fish growth, immunity, and intestinal health. Intestinal health is especially important for carnivorous fish such as rainbow trout (*Oncorhynchus mykiss*), which have a relatively small intestinal length index. In this study, it was confirmed that dietary NaB levels of 0.20% improved the growth of juvenile *O. mykiss* and reduced the severity of anti-nutritional factor-induced enteropathy by enhancing digestive enzymes and improving intestinal morphology. Dietary NaB enhanced the mRNA expression of TJ proteins while decreasing the mRNA expression of pro-inflammatory cytokines, NF-κB, and MLCK-MLC, likely reinforcing the intestinal mucosal barrier and protecting against pathogenic bacterial infections. In addition, the gut microbial composition was more conducive to *O. mykiss* health with the inclusion of 0.20% NaB in the diet. Furthermore, when challenged with *Aeromonas salmonicida*, dietary 0.20% NaB increased the survival rate of *O. mykiss*. This study provides a theoretical basis for the development of feed additives for use in low-fish meal feeds.

**Abstract:**

This study aimed to determine the effects of dietary sodium butyrate (NaB) on the growth and gut health of triploid *Oncorhynchus mykiss* juveniles (8.86 ± 0.36 g) fed a low fish meal diet for 8 weeks, including the inflammatory response, histomorphology, and the composition and functional prediction of microbiota. Five isonitrogenous and isoenergetic practical diets (15.00% fish meal and 21.60% soybean meal) were supplemented with 0.00% (G1), 0.10% (G2), 0.20% (G3), 0.30% (G4), and 0.40% NaB (G5), respectively. After the feeding trial, the mortality for G3 challenged with *Aeromonas salmonicida* for 7 days was lower than that for G1 and G5. The optimal NaB requirement for triploid *O. mykiss* based on weight gain rate (WGR) and the specific growth rate (SGR) was estimated to be 0.22% and 0.20%, respectively. The activities of intestinal digestive enzymes increased in fish fed a NaB diet compared to G1 (*p* < 0.05). G1 also showed obvious signs of inflammation, but this inflammation was significantly alleviated with dietary NaB supplementation. In comparison, G3 exhibited a more complete intestinal mucosal morphology. Dietary 0.20% NaB may play an anti-inflammatory role by inhibiting the NF-κB-P65 inflammatory signaling pathway. Additionally, the relative abundance of probiotics was altered by dietary NaB. In conclusion, dietary 0.20% NaB improved the intestinal health of triploid *O. mykiss* fed a low fish meal diet.

## 1. Introduction

The rapid growth of aquaculture in recent years has resulted in a global deficit of fish meal supplies, which has prompted the urgent need for alternative protein sources for fish meal [1]. Plant protein is considered a crucial alternate protein source to fish meal, but it contains antinutritional elements and poisonous compounds, such as cotton phenol that can cause intestinal damage [2,3,4]. Soybean meal is currently one of the most widely used ingredients in aquafeed due to its consistent supply, fair price, and well-balanced amino acid profile [5]. However, anti-nutritional factors (ANF) such as antigen protein (AP) and soybean agglutinin (SBA) in soybean meal can also cause intestinal barrier injury and inflammatory reactions in fish. This can harm fish growth, immunity, and intestinal health [6,7,8,9,10]. The intestinal barrier is a complex combination of interactions between the immune cells, epithelial barrier, and gut microbiota [11]. Intestinal health is especially important for carnivorous fish such as rainbow trout (*Oncorhynchus mykiss*), as they have a relatively small intestinal length index. Therefore, functional feed additives that can reduce intestinal injury caused by ANF in plant proteins have yet to be developed. Several functional ingredients, such as glutathione and arginine, have been shown to protect intestinal health in *O. mykiss* fed a high level of dietary soybean protein [12,13].

Short-chain fatty acids (SCFAs), particularly butyric acid (BA), play a crucial role in maintaining the integrity of the intestinal epithelial barrier [14]. BA influences the functions of various host systems, including the intestinal, hematological, neurological, and endocrine systems, and it has anti-inflammatory effects that are closely linked to intestinal inflammation [15]. In addition to being absorbed by transporter proteins such as MCT1 and SMCT1 to support cellular metabolism, BA can act as a signaling molecule [16]. Previous in vivo and in vitro studies of NaB have demonstrated its ability to suppress NF-κB, leading to a decrease in the expression and production of pro-inflammatory cytokines and the innate immune response [17,18,19,20]. Lower concentrations of BA (up to 2 mM) have been shown to reduce permeability in HT-29 and Caco-2 cell lines [21,22], but higher concentrations (8 mM and 10 mM) significantly increased the permeability of Caco-2 cell lines and the distal colonic mucosa of adult rats [21,23]. Additionally, previous research has also shown that dietary NaB may be used to protect aquatic creatures against enteropathy (enteritis) by activating antioxidant defense responses and enhancing local immunity in the intestine against invading pathogenic organisms.

The gut microbial composition is critical for growth, and a stable and rational composition of the microbial community is important for fish health. Rawls et al. found that the gut of zebrafish (*Danio rerio*) has 212 genes related to the microbial community in the intestine [24]. The gut microbial community performs various functions, such as stimulating epithelial cell renewal [25], promoting nutrient metabolism [26], and enhancing immune function [27]. By studying the intestinal microbiota of fish, researchers can use diet to manipulate the number of intestinal probiotics and improve fish productivity [28]. Dietary NaB has been shown to alter the composition of the gut microorganisms in turbot (*Scophthalmus maximus* L.), which were more similar to the fish meal (FM) group than the soybean meal (SBM) group [29]. However, research on common carp (*Cyprinus carpio*) has shown that NaB has no significant effect on the microbial populations in the intestine [30]. However, research on the active mechanism of BA on the gut microbiota of carnivorous fish is limited. It is unclear why NaB affects the gut microbiota of different dietary fish in different ways.

According to the Food and Agriculture Organization of the United Nations (FAO) [31], farmed salmon and trout production exceeds 3 million tons annually, making it the third largest aquaculture species in the world as of 2020. In recent years, trout farming in China has grown rapidly and has become one of the main cold-water fish farming species in the country, with annual production reaching 30,000 tons [32]. *O. mykiss* has been cultivated in China since the 1950s, and its triploids can now be found in most provinces and regions of the country. Triploid *O. mykiss* are poorly fertile, which means that they do not use all of their energy for growth. However, they grow faster than diploid *O. mykiss* during the gonadal maturity stage, and they have better disease resistance and flesh quality [33]. This study aimed to investigate the effects of dietary NaB on growth, digestion, intestinal histology, inflammation, histomorphology, microbiota composition and function, and resistance to *Aeromonas salmonicida* in juvenile triploid *O. mykiss* (8.86 ± 0.36 g) fed a low fish meal diet. By evaluating the relative expression of genes involved in the NF-κB and MLCK-MLC signaling pathways, we examined the effects of dietary NaB on intestinal cell inflammation and mucosal permeability. This work serves as a reference for further research on the process by which BA restores intestinal mucosa. To determine whether NaB affects the gut microbiota of *O. mykiss*, we analyzed the hypervariable V4 region of bacteria using an Illumina MiSeq sequencer. These findings will provide a foundation for future studies on the intestinal immune system of triploid *O. mykiss* and the development of low-fish meal compound diets.

## 2. Materials and Methods

### 2.1. Diets

In this experiment, five groups were identified: the control group (G1) was fed a basal diet, and four additional groups (G2–G5) were each supplemented with a different amount of NaB at concentrations of 0.10%, 0.20%, 0.30%, and 0.40%, respectively. The composition of the basal diet is listed in Table 1. Wheat flour, fish meal, and soybean meal were used as carbohydrate, protein, and lipid sources, respectively. The ingredients were weighed, finely ground, and blended according to the specified formulations. Lipid was added, and the materials were mixed for 25 min. The mixture was then homogenized again by adding a sufficient amount of distilled water to reach a caking state. Using a pelletizer (HX-200G; Guanghe Instruments, Guangzhou, China), the mixture was formed into 1 mm pellets, which were air-dried at 60 °C, cooled to room temperature, and stored in sealed bags in a −20 °C refrigerator. 

### 2.2. Experimental Conditions

The *O. mykiss* used in this experiment were purchased from Agrimarine Holdings Inc. (Benxi, China) and acclimated by being fed a basal diet for 14 days before the start of the experiment. From a pool of 450 fish with an initial weight of 8.86 ± 0.36 g, 15 tanks (each with a volume of 500 L) containing 30 fish each were randomly chosen. The fish were fed the test diets until apparent satiation twice a day during the 8-week feeding trial, which took place at 8:00 a.m. and 4:00 p.m. The fish were weighed every 14 days to determine body weight and adjust meal amounts. The amount of feed administered per week was counted, and the total feed amount was calculated. The *O. mykiss* were kept in a recirculating aquaculture system with a temperature control system and water filtration system. The water was aerated tap water, which was controlled to have the following parameters: temperature 14 ± 0.5 °C, dissolved oxygen > 6.0 mg/L, pH 6.8–7.1, NO^2™^-N < 0.02 mg/L, and NH^4+^-N < 0.20 mg/L. The water quality was measured using a YSI 8500 SELECT™ Biochemistry Analyzer (YSI Inc., Yellow Springs, OH, USA), and approximately 160 L of water per tank was renewed daily to ensure suitable water quality.

### 2.3. Experimental Sample Collection

At the end of the 56-day feeding trial, *O. mykiss* were fasted for one day before sampling to empty the contents of their digestive tracts. They were then weighed after being anesthetized with MS-222 (75 mg/L), and the total weight of each tank was recorded to calculate the growth index using a balance (ME204E, METTLER TOLEDO Technology Co., Shanghai, China). Tail vein blood samples were taken, and the supernatant was collected as serum after centrifugation at 1530× *g* for 10 min at 4 °C. The serum was kept at −40 °C for subsequent biochemical assays. Six *O. mykiss* were used to obtain mid-intestine samples (between the pyloric and hindgut), which were kept at −80 °C for biochemical examination. Three different fish were dissected to obtain proximal intestine samples, which were immediately frozen in liquid nitrogen and stored at −80 °C for gene expression analysis. Two *O. mykiss* were sterilized with 75% alcohol, and the whole mucosa layer of the distal intestine was removed using sterile tools under an alcohol lamp for the investigation of the intestinal microbiota. Afterward, the intestines of three fish from each group were removed, the contents were cleared, and the fish were placed in Bouin’s solution for intestinal histology.

### 2.4. Nutrient Proximate Analysis

The nutrient levels of the whole *O. mykiss* and test diets were determined according to the standard methods described in AOAC (2012) [34]. The samples’ moisture content was determined by baking them at 105 °C for three hours. The Kjeldahl method (method 2001.11) was used to measure nitrogen in order to assess crude protein (N × 6.25; 2300, FOSS Tecator AB, Hogans, Sweden). The ash concentration was determined by burning the samples for two hours at 600 °C (method number: 942.05). Crude lipid was extracted from the *O. mykiss* fishmeal using the Soxhlet extraction method (method number: 920.39).

### 2.5. LPS, AMS, and Trypsin Activities

The activities of intestinal lipase (LPS), amylase (AMS), and trypsin were measured using commercially available kits (Nanjing Jiancheng Bioengineering Institute, Nanjing, China). The intestinal tissue homogenates were centrifuged at 861× *g* for 10 min after adding 9 volumes (*w/v*) of 5.3% ice-cold saline. The supernatant was then centrifuged and stored at −80 °C until needed. Lipase (LPS; A054-2-1) activity was measured at 580 nm based on the rate of production of methyl tachykinin in red. The amount of hydrolyzed starch was calculated based on the blue complex formed when the unhydrolyzed starch was mixed with iodine solution and its absorbance at 660 nm, allowing for the calculation of intestinal amylase (AMS; C016-1-1) activity. The UV colorimetric approach was used to determine the amount of trypsin (A080-2-2) present. Total protein (TP) was quantified using the Coomassie bright blue method at 595 nm. All absorbance measurements were performed using a microplate reader (Synergy 2, BoiTek Instruments, Inc., Winooski, VT, USA).

### 2.6. Histological Examination

Four *O. mykiss* intestinal segments without alimentary canal contents were randomly placed in Bouin’s solution, rinsed multiple times in water, and soaked in 70% ethanol until the ethanol did not change color. The dehydrated midguts were then embedded in normal paraffin. Using a microtome (HistoCore MULTICUT, Leica, Shanghai, China), the material was cut into 4 μm thick sections. After deparaffinization and hydration, the slides were stained with hematoxylin and eosin and mounted with neutral resin. Each group had 30 sections of the MI examined under a microscope (Echo Revolve, San Diego, CA, USA).

### 2.7. Gene Expression Analyses

Liquid nitrogen was used to grind *O. mykiss* gut samples that were stored at −80 °C. Total RNA was extracted from the resulting powder using TRIzol Reagent (Ambion, San Diego, CA, USA) and then reverse transcribed into cDNA (RR047A; TaKaRa, Beijing, China). The quality of the RNA and cDNA was assessed using agarose gel electrophoresis (visible bands) and a Thermo Scientific NanoDrop 2000 UV-Vis spectrophotometer (A260 nm/A280 nm: 1.8–2.0). cDNA was used as template DNA for RT-PCR amplification reactions. Three replicates of each amplification reaction were used for comparison (Table 2). Table 3 lists the sequences of all the primers used in this study. The internal reference gene β-actin was used to normalize cDNA loading. The 2^−ΔΔCT^ method was used to compare the expression results of each gene mRNA [35].

### 2.8. 16S rRNA Gene Sequencing and Bioinformatic Analysis

The purity of total microbial DNA extracted from the alimentary canal contents of *O. mykiss* was assessed using A230 nm, A260 nm, and A280 nm. The highly variable V3-V4 region of the bacterial 16S rRNA gene was amplified according to the PCR procedure in Table 2. The PCR amplification products were purified using agarose gel electrophoresis (AxyPrep DNA Gel Extraction Kit; Axygen Biosciences, Union City, CA, USA).

Illumina adapter sequences were added to the outer end of the target region by PCR and used to construct the Miseq library (Illumina, Shanghai, China). Pair-end sequencing was performed on Miseq technology. The PE reads obtained from Miseq sequencing were first spliced according to the overlapping relationship (Fast Length Adjustment of Short Reads, FLASH 1.2.11) [36], while sequence quality was quality-controlled (Uparse 11) and filtered (Usearch 11), and operational taxonomic units (OTUs) clustering analysis and species taxonomy analysis were performed after differentiating samples. Based on the results of OTU clustering analysis, various diversity indices and the detection of sequencing depth for OTUs can be analyzed, such as alpha diversity analysis (Mothur 1.30.2) and beta diversity analysis (Qiime 1.9.1) [37]. UCHIME was used to identify chimeric sequences [38]. The raw reads were submitted to the NCBI Sequence Read Archive (SRA, accession number: PRJNA874478). A statistical analysis of community structure can be performed at each taxonomic level using the taxonomic information. Based on the above analysis, a series of in-depth statistical and visualization analyses, such as multivariate analysis and significance tests, can be performed on the community composition and phylogenetic information of multiple species.

Principal component analysis (PCA) plots based on the OTU level were used to assess beta diversity. Different intestinal bacteria were found in each of the five groups, and the microbial makeup at the phylum and genus levels was examined. To identify which bacterial taxa were different between groups, a linear discriminant analysis (LDA) effect size (LEfSe) investigation was carried out using the Python LEfSe module [39]. Non-binary co-expression network analysis was used to measure the intra-order interaction of variables (weighted topological, wTO). The wTO technique calculates a link-to-link threshold of significance with a *p*-value adjusted for multiple tests of 0.05 [40]. Phylogenetic Investigation of Communities by Reconstruction of Unobserved States (PICRUSt2) was used to predict functional pathways in KEGG Orthology (KOs) [41] using the 16S rRNA gene sequencing data. The phenotypic characteristics of the bacterial community were predicted using BugBase (http://bugbase.cs.umn.edu accessed on 3 November 2017).

### 2.9. Aeromonas Salmonicida Challenge

After sampling, the experimental fish were fed test diets for 7 days. Sixty fish from each group were randomly separated into three tanks, with 20 fish from each tank examined for *A. salmonicida* infection. Bacteria were incubated for 14 h at 28 °C in trypticase soy broth (TSB). *A. salmonicida* was diluted to 1.08 × 10^8^ CFU/mL (LD_50_) in phosphate-buffered saline (PBS), and 100 μL of the bacteria suspension was injected into the base of the pectoral fin of each fish. The number of dead fish was recorded every 24 h for 7 days after *A. salmonicida* infection and starvation.

### 2.10. Calculations

The growth performance parameters were calculated as follows:

weight gain rate (WGR; %) = 100 × (W_56_ − W_0_)/W_0_;
specific growth rate (SGR; %/d) = 100 × (lnW_56_ − lnW_0_)/56 days;
protein efficiency ratio (PER) = (W_56_ − W_0_)/ (W_f_ × feed protein content);
feed conversion ratio (FCR) = W_f_/ (W_56_ − W_0_);
hepatosomatic index (HSI; %) = 100 × (liver weight (g)/body weight (g));
condition factor (CF; %) = 100 × W_56_/L_56_^3^;
viscerosomatic index (VSI; %) = 100 × (viscera (g)/body weight (g));
survival rate (SR, %) = 100 × W_56_/W_0_,
where W_0_ represents the initial body weight (g), W_56_ represents the final body weight (g), W_f_ represents the feed intake (g), and L_56_ represents the final body length (cm).

A one-way analysis of variance, a covariance analysis, and Tukey multiple comparisons were conducted on the data, and the group and initial weight were tested for their effect on the WGR and SGR using covariance analysis (SPSS 20.0, SPSS Inc., Chicago, IL, USA). The data showed substantial variations within the group, as indicated by a *p*-value of less than 0.05. The mean and standard error (S.E.) were used to express the data, and a broken-line analysis was performed using Graphpad Prism 8.0 to estimate the optimum range of NaB requirements for triploid *O. mykiss* under low fish meal conditions based on the WGR and SGR.

## 3. Results

### 3.1. Growth and Feeding Parameters

**Figure 1:**
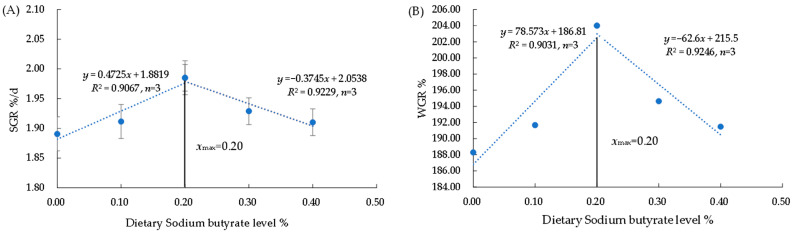
Regressive analysis between dietary NaB level and SGR of *O. mykiss* (**A**). Regressive analysis between dietary NaB level and WGR of *O. mykiss* (**B**).

There was no significant difference in SR and CF among the groups (*p* > 0.05) (Table 4). The WGR, SGR, and PER were significantly higher in the G3 group compared to the G1 group (*p* < 0.05). The value of FCR was significantly lower in the G3 group (*p* < 0.05). The WGR and SGR rose and then declined as dietary NaB levels increased, reaching a maximum in the 0.20% group and being significantly higher than those of the control group (*p* < 0.05) (Figure 1). Based on the broken-line analysis of SGR and WGR, the optimal NaB requirement for triploid *O. mykiss* was 0.20%.

Table 4 demonstrates that all four treatment groups had significantly lower HSI and VSI than G1 (*p* < 0.05).

### 3.2. Body Composition

As shown in Table 5, dietary NaB did not affect whole-body moisture, crude lipid, crude protein, and ash levels (*p* > 0.05).

### 3.3. Digestive Physiology

Table 6 shows the impact of varied dietary NaB levels on the intestinal digestive enzyme activity of triploid *O. mykiss*. The activity of trypsin, LPS, and AMS was significantly higher in the 0.20% group (G3) compared to the other groups (*p* < 0.05). These enzyme activities then decreased gradually as the NaB level increased. LPS activity was also found to be lower in the 0.40% group (G5) compared to the control group (*p* < 0.05).

### 3.4. Histology

According to the data in Table 6, adding NaB to the diet significantly increased the values of villi height, villi width, and muscular thickness of the *O. mykiss* midgut (*p* < 0.05). The largest values of these parameters were observed when NaB was added at 0.20% levels (G3). However, dietary 0.40% NaB resulted in significantly lower villi height and villi width compared to the control group (*p* < 0.05).

**Figure 2:**
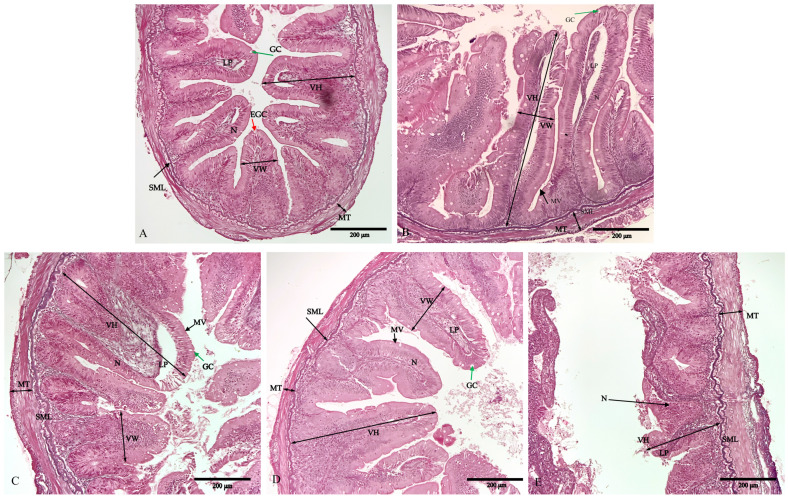
Effects of dietary sodium butyrate level on intestinal morphology of triploid *O. mykiss*. Details of the distal intestine section from fish in the G1 (**A**), G2 (**B**), G3 (**C**), G4 (**D**), and G5 (**E**) groups. VH: villi height; VW: villus width; MT: muscle layer thickness; SML: submucous layer; LP: lamina propria; GC: goblet cell (green arrows); MV: microvilli (arrowheads); N: nucleus; EGC: eosinophilic granular cell (red arrow). Scale bar: 200 μm.

The intestine in the control group was structurally incomplete, with absorptive vacuoles of epithelial cells in simple mucosal foldings that were round-shaped and irregularly arranged, a loss of tissue microvilli, and blurred morphological contours of the striated border (Figure 2). The intestine also showed obvious signs of inflammation, including wizened enterocytes, enterocytes with disordered nuclei, and mixed leukocyte infiltration in the lamina propria. In contrast, the diets supplemented with 0.20% and 0.30% NaB (plates C and D) had the healthiest *O. mykiss* intestinal histomorphology among the five groups, with obvious goblet cells and non-vacuolated areas, well-developed microvilli arranged in an orderly fashion without fusion or exfoliation, a significant increase in the number of goblet cells, and a compactly arranged simple columnar epithelium.

### 3.5. Intestinal Gene Expression

**Figure 3:**
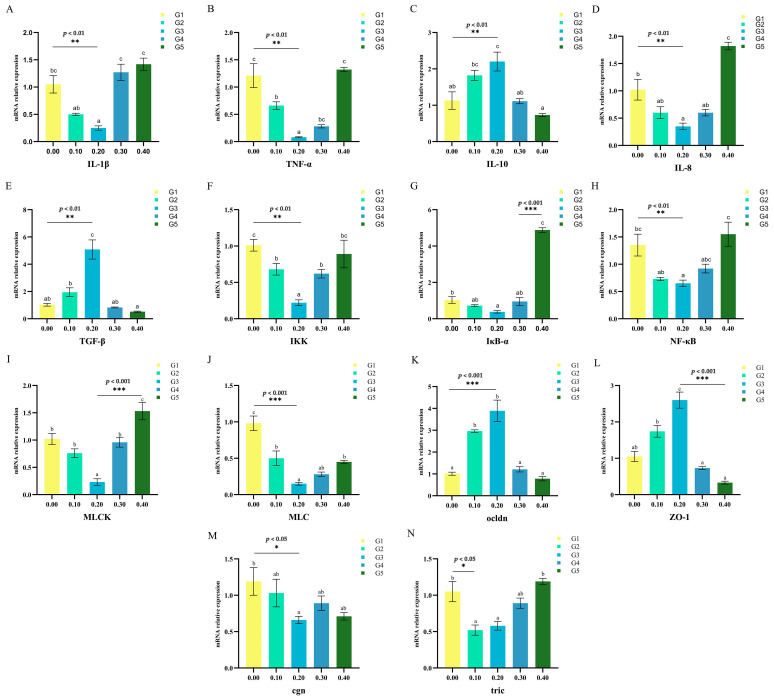
Effects of NaB on the expression of IL-1β (**A**), TNF-α (**B**), IL-10 (**C**), IL-8 (**D**), TGF-β(**E**), IKK (**F**), IκB-α (**G**), NF-κB (**H**), MLCK (**I**), MLC (**J**), ocldn (**K**), ZO-1 (**L**), cgn (**M**) and tric (**N**) in intestinal mucosal barrier of juvenile triploid *O. mykiss*. ^abc^ different superscript letters are significantly different (*p* < 0.05). All error bars represent S.E.; * *p* < 0.05, ** *p* < 0.01 and *** *p* < 0.001.

Gene expression analysis was performed on intestinal samples collected from *O. mykiss* fed low fish meal diets. It was found that the addition of NaB at a concentration of 0.20% caused a significant decrease in the expression of IL-1β, IL-8, IKK, NF-κB, IκB-α, TNF-α, MLCK, and MLC genes in the intestine of *O. mykiss* (*p* < 0.05) (Figure 3).

### 3.6. Gut Bacterial Community Composition

**Figure 4:**
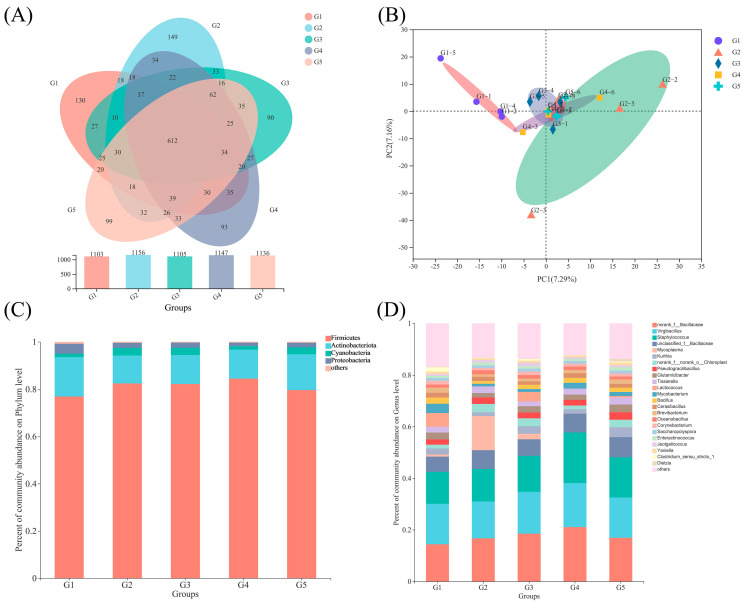
Venn diagram showing the number of shared and unique OTUs in the five groups (**A**). The OUT level (**B**) is used for principal component analysis (PCA). Relative abundance of intestinal microbes in *O. mykiss* at the phylum (**C**) and genus level (**D**).

The Illumina MiSeq PE300 reading system generated an average of 40,862 sequences per sample from the 30 sequenced samples, totaling 530,999,267 effective sequences after deleting low-quality reads and chimeras. The sequences ranged in length from 212 to 516 bp, with an average length of 424 bp. The percentage of good coverage for each group exceeded 99.9%, demonstrating the accuracy and reliability of the diverse results. The rarefaction curves reached a plateau, indicating that the sequencing depth was sufficient to represent the diversity of the microbial species. The similarities and overlaps of OTUs (not defined) in the various groups were evaluated using a Venn diagram. The five groups shared 612 OTUs, and each group had a unique number of OTUs: 130 (G1), 149 (G2), 90 (G3), 93 (G4), and 99 (G5) (Figure 4A).

Furthermore, the beta diversity analysis based on weighted and unweighted UniFrac distances indicated that the five groups of gut bacteria were separated and grouped in different sections of the PCA plot (Figure 4B). According to (Figure 4B), *O. mykiss* in the various treatment groups had significantly different gut microbial compositions, with the PC1 and PC2 axes explaining 7.16% and 7.29%, respectively.

There were 14 distinct bacterial phyla identified. At the phylum level, the relative abundance of Firmicutes was lower in the G1 group compared to the other four groups, while Actinobacteriota and Proteobacteria had higher relative abundances (Figure 4C).

**Figure 5:**
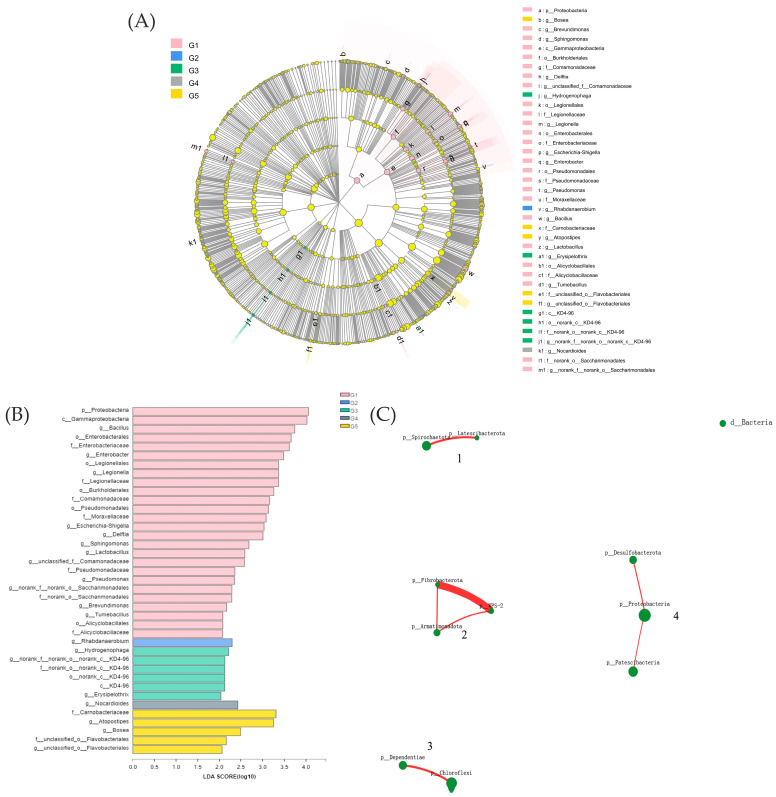
Inter-group variation in the relative abundance of intestinal microbial communities (**A**). LDA score from Lefse-PICRUSt (**B**). Network interaction graph for the hindgut microbial communities at the order level, using weighted topological (wTO) network analysis (**C**). Note: Red-colored links are positive correlations, while negative correlations are in green-colored links. The thickness of the line indicates the magnitude of the correlation coefficient.

We conducted LEfSe analysis (Figure 5A) on the five groups and identified several bacterial genera as biomarkers of group difference: *Bacillus*, *Enterobacter*, *Legionella*, *Escherichia-Shigella*, *Delftia*, *Sphingomonas*, *Lactobacillus*, *Pseudomonas*, *Brevundimonas*, *Tumebacillus*, and others. Proteobacteria were the phylum-level biomarkers in G1, while *Rhabdanaerobium* was the genus-level biomarker in G2. *Hydrogenophaga*, nor-ank-f-norank-o-norank-c-KD4-96, and *Erysipelothrix* were the genus-level biomarkers in G3. *Nocardioides* were the genus-level biomarkers in G4, and *Atopostipes* and *Bosea* were the genus-level biomarkers in G5 (Figure 5B).

**Figure 6:**
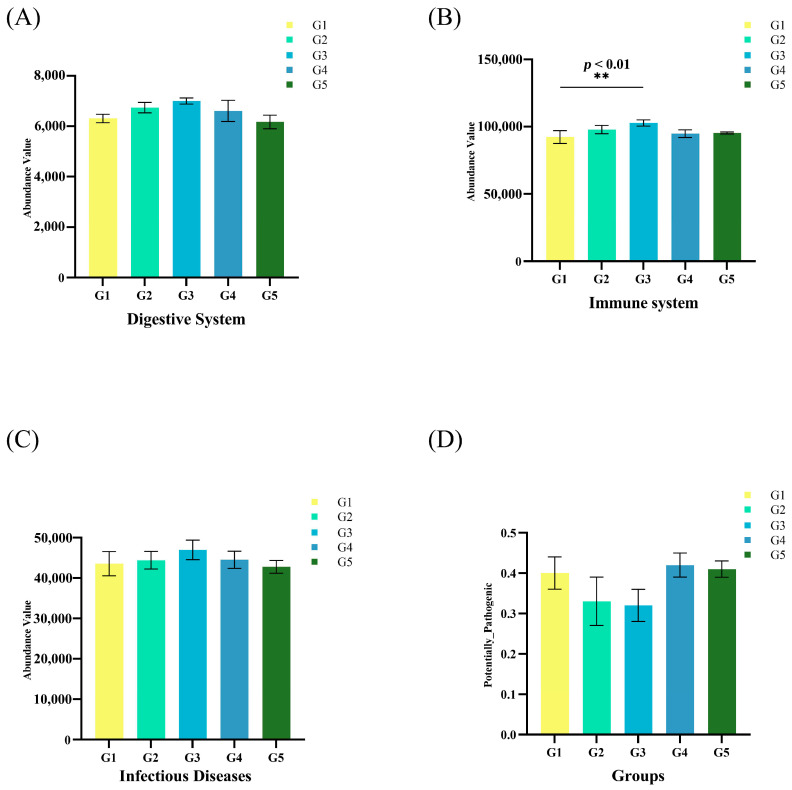
Predictive metagenomic analysis of *O. mykiss* fecal microbiota functional profiling. Bacterial gene functions were predicted from the 16S rRNA gene-based microbial compositions using the PICRUSt2 algorithm and inferences from KEGG databases. Abundance values of the digestive system (**A**), immune system (**B**), and infectious diseases (**C**) in the gut microbiota of *O. mykiss*. Relative abundance values of potential pathogenicity in the gut microbiota of *O. mykiss* (**D**). All error bars represent S.E.; ** *p* < 0.01).

The gut microbiota was divided into four clusters, all of which had primarily positive interactions. The majority of the microbes in Cluster 4 were from the Proteobacteria phylum, while those in Cluster 1 came from the Spirochaetota phylum. The Fibrobacteria phylum and the WPS-2 phylum had the highest positive correlation and a symbiotic relationship (Figure 5C). The lowest relative abundance values for potential pathogenicity were predicted in G3, according to BugBase results for triploid *O. mykiss* gut microbiota, but these values were not significantly different from those of the other four groups (Figure 6D). Using the PICRUSt2 algorithm and deductions from the KEGG databases, the 16S rRNA gene-based microbial compositions were used to estimate bacterial gene functions. The abundance of the G3 immune system was significantly greater than that of the other four groups (*p* < 0.05) (Figure 6B). There were no significant differences in the abundance values for the digestive system and disease resistance among the five groups, but G3 had the highest values (Figure 6A,C).

### 3.7. Survival against the A. salmonicida Challenge

**Figure 7:**
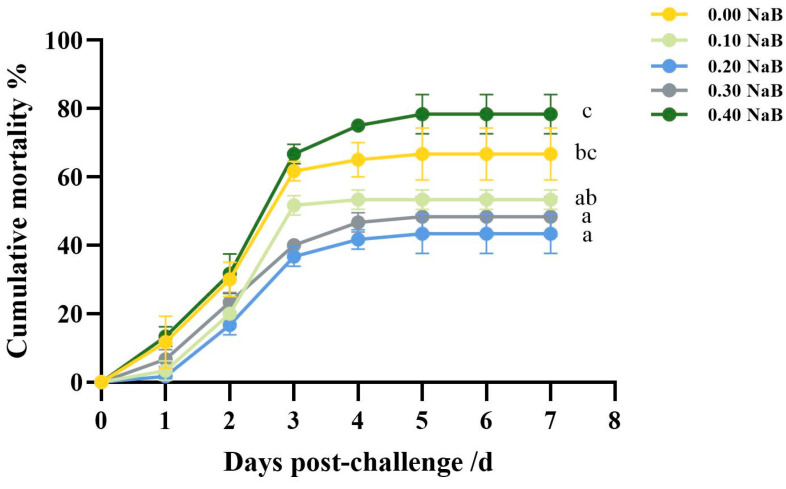
Effect of NaB supplementation on survival against bacterial infection. Different superscript letters indicate significant difference (*p* < 0.05).

After being challenged with *A. salmonicida*, fish that were given a diet supplemented with 0.20% NaB (G3) had a lower average mortality rate of 33.33% compared to the 66.67% rate for fish fed a non-supplemented diet (G1) and the 78.33% rate for fish fed a diet supplemented with 0.40% NaB (G5). The death occurred from the first to the fifth day following *A. salmonicida* inoculation (Figure 7).

## 4. Discussion

### 4.1. Growth Parameters

The optimal amount of NaB to add varies by species and has different effects on its growth performance. Elmazni et al. found that 0.05% NaB significantly improved the body weight of male New Zealand rabbits [42]. Diets with 1.00% and 2.00% NaB significantly improved the survival and weight gain of juvenile Chinese mitten crabs (*Eriocheir sinensis*) compared to those in the 0% NaB group [43]. The inclusion of 0.117% NaB in diets for *Arapaima gigas* juveniles improves growth parameters [44]. The equation for the relationship between the concentration of NaB added (*x*) and WGR (*y*) was obtained by fitting a linear, quadratic, and cubic model: y = 78.573 x + 186.81 (R^2^ = 0.9031), y = −248.56 x^2^ + 108.77 x + 187.18 (R^2^ = 0.6578), y = −227.07 x^3^ − 112.32 x^2^ + 89.245 x + 187.46 (R^2^ = 0.6629). The equation for the relationship between the concentration of NaB added (x) and SGR (y) was obtained by fitting a linear, quadratic, and cubic model: y = 0.4725 x + 1.8819 (R^2^ = 0.9067), y = −1.93 x^2^ + 0.7674 x + 1.8815 (R^2^ = 0.6629), y = −1.25 x^3^ − 0.7436 x^2^ + 0.5464 x + 1.8856 (R^2^ = 0.6652). Among them, the R^2^ value of the linear equation was the highest. However, this study used the linear regression method and the SGR and WGR as evaluation indices to calculate that the NaB requirements for juvenile triploid *O. mykiss* were 0.20% (Figure 1).

Liu et al. found that adding either 0.10% or 0.20% NaB to the diet significantly increased the SGR of grass carp (*Ctenopharyngodon idella*) [45]. It was also discovered that adding 0.20% NaB to the diet significantly altered the immune-related gene expression and improved the immunity of European seabass (*Dicentrarchus labrax*) without significantly affecting the WGR and SGR of the fish [46]. This study found that adding 0.20% NaB to the diet increased the weight gain, feed efficiency, and SGR of juvenile *O. mykiss*. This may be because NaB is a direct energy source for intestinal cells. When it enters the intestine, it can stimulate the rapid repair of damaged villi by renewing intermediate cells, making the villi longer, and improving the ability to digest and absorb nutrients. The addition of NaB also increases the ability of juvenile *O. mykiss* to absorb nutrients and strengthens its antioxidant defenses. Additionally, NaB can promote the proliferation and maturation of intestinal cells, improve the morphology and barrier function of the intestine, and have bactericidal, antibacterial, and anti-inflammatory effects on the intestine, helping to maintain intestinal health and improve growth performance.

The addition of excess NaB in this study suppressed the growth of *O. mykiss*, which may be due to the low pH in the intestine caused by the excess NaB, which inhibits the functioning of neutral and alkaline digestive enzymes. Additionally, excessive amounts of BA prevented the normal development of the digestive tract in *O. mykiss* and made nutrients in the intestine less digestible. Zhang et al. found that NaB did not affect feed palatability and that its growth-promoting effect on fish was achieved not by increasing feeding amounts, but by improving feed utilization [47].

### 4.2. Body Composition

A previous study found that the whole-body composition of grass carp was not affected by dietary NaB [45]. Similarly, this experiment found that the crude protein, crude lipid, and ash levels of juvenile triploid *O. mykiss* were not affected by dietary NaB supplementation. However, it was discovered that dietary NaB significantly enhanced the crude protein content of whole tilapia (*Oreochromis mossambicus*). This is likely due to NaB promoting a more efficient conversion of ingested food into structural proteins, which increases fish muscle production, although the exact mechanism is not known [48].

### 4.3. Digestive Physiology and Histological Analyses

Poor-quality protein sources can significantly reduce the height of the intestinal mucosa, blur epithelial cell borders, or even cause separation, which limits the utilization of protein sources and hinders the ability of fish to grow healthily [49,50]. Therefore, it is important to protect the intestinal health of the fish. Zhou et al. found that *Trachinotus ovatus* had significantly higher intestinal protease, amylase, and alkaline protease activities when 2.0 and 4.0 g/kg of NaB were added to its diet [51]. Aalamifar et al. found that adding 5.0 or 10.0 g/kg of NaB to the feed of *Latescalcarifer* significantly increased the overall intestine alkaline protease and lipase activities compared to the control group [52]. Similar results were found with triploid *O. mykiss*, where the addition of more NaB initially caused an increase and then a decrease in the activity of intestinal digestive enzymes. *O. mykiss* has a shorter digestive system, fewer active digestive enzymes, and a shorter feed residence time in the colon than omnivorous fish. The main purpose of adding NaB to low fish meal feeds is to improve fish digestion by promoting the production of digestive enzymes and improving gut absorption, which will improve fish growth and development [53].

The surface area of intestinal absorption for carnivorous fish such as *O. mykiss*, which have relatively short intestinal length indices, is closely related to their ability to digest and absorb nutrients. Therefore, the expansion of the microvilli and the increased height and width of the intestinal villi can enhance the absorption area of the intestine, improving the efficiency of nutrient absorption [54]. The addition of 0.20% NaB to the diet of low fish meal *D. labrax* for 60 days reduced the inflammatory response in the posterior segment intestine and restored normal morphology [50]. When 0.20% microencapsulated NaB was added to the diet, it accelerated the proliferation of intestinal epithelial cells, increased the length and density of the villi, and encouraged goblet cell mucus production, all of which helped to maintain the development and integrity of the gut morphological structure [55]. The addition of 0.05% NaB to the feed of *C. idella* significantly increased the height of the intestinal villi [56]. Low fish meal *A. gigas* diets supplemented with 0.20% NaB for 12 weeks improved intestinal pathology, including an increase in the intestinal villi absorption surface area, a reduction in the inflammatory infiltration of intestinal leukocytes, and an increase in enzyme activity in the brush border of the intestine [44]. This study obtained similar results, and the potential mechanism by which dietary NaB improves intestinal morphology, digestion, and absorption is by providing energy to the intestinal epithelial cells. This can stimulate normal intestinal epithelial cell development and enzyme production that aids in mucosal repair and intestinal mucosa repair. NaB also stimulates intracellular mRNA and protein synthesis, increases villi height, promotes digestion and absorption, and improves growth performance.

### 4.4. Intestinal Microbial Diversity Analyses and Functional Prediction

The regular intestinal flora of fish is essential for their growth and development because it not only helps with nutrient digestion and absorption from the intestines but also helps the fish maintain its overall health by activating its immune system [57]. The operation of the intestinal barrier is directly linked to the gut bacteria and their metabolites. The findings of this study, which are in line with earlier research on fish or terrestrial animals, showed that the gut bacterial compositions of fish are highly responsive to diets [58,59,60]. In this study, α-diversity, Sobs, Ace, and Chao significantly increased with increasing dietary NaB concentrations. NaB has also been shown to alter the composition of the intestinal microbiota and increase the diversity of the intestinal microbiota in tests with carp (*Cyprinus carpio*) and gilthead sea bream (*Sparus aurata*). However, adding 0.40% NaB was able to prevent developmental delays in parasitized fish and increase the diversity of the gut microbiota by increasing the number of bacteria that produce butyrate [61]. In comparison to humans and other mammals, the potential mechanisms of NaB on fish gut microbiota are largely unknown. This may be because NaB enters bacterial cells as non-dissociated butyric acid, which then dissociates into CH_3_-CH_2_-CH_2_-COO^™^ and H^+^. Harmful bacteria such as *Escherichia coli* and *Salmonella*, which are poorly tolerant of H^+^, die in large numbers, while helpful bacteria such as *Lactobacillus* and *Bifidobacterium*, which are highly tolerant of H^+^, proliferate [62]. One type of organic acid, BA, decreases the pH in the fish digestive tract and prevents the overgrowth of harmful bacteria that are sensitive to pH [63]. Furthermore, based on the Venn diagram results and the α-diversity of the gut microbiota, it is possible to conclude that the addition of 0.2% NaB (G3) resulted in a healthier microbial composition and structure compared to G1, indicating that NaB positively contributes to the maintenance of the balance between the various microbial populations in the intestine. Previous studies have shown that probiotics in the gut produce enzymes that help with digestion and nutrient absorption [64]. This may be an important mode of interaction between the host and the indigenous microbiota [65]. *Proteobacteria*, for example, can utilize external environmental reservoirs to proliferate and reach a relatively high prevalence in fish guts [25,66]. Bacteria from the *Actinobacteria* genus produce secondary metabolites and extracellular enzymes [67]. The relative abundance of *Proteobacteria* and *Actinobacteria* was significantly reduced (G1) when 0.20% NaB was added to the diet. In the phylum Bacteroidetes, *Bacteroides* can secrete enzymes for breaking down and metabolizing sugars and polysaccharides [58,68]. The increased population of helpful bacteria in the gut helps *O. mykiss* digest plant proteins.

In the long term, there were differences in the predicted functional profiles between gut tract fractions. The nutrition and immunity of the host may be affected by the gut microbiota. According to the microbial makeup and functional capabilities, the five groups in this investigation varied in their predictions of functional pathways. Our findings showed that the diet containing 0.20% NaB had higher levels of genes related to the immune system and the digestive system (G3). Furthermore, *O. mykiss* used protein as a source of energy for growth. The gut microbiota, on the other hand, have a digestive system that may boost metabolic power and increase the abundance of the Firmicutesto phylum to compensate for the host deficiencies. Because dietary NaB significantly reduced the abundance of the Proteobacteria phylum in *O. mykiss* gut microorganisms, the gut microbiota exhibited immune responses. The lowest relative abundance values for potential pathogenicity were predicted in the 0.20% NaB-supplemented diet (G3) based on BugBase results for triploid *O. mykiss* gut microbiota. This was also an important factor that adding NaB can improve the immunity and disease resistance of *O. mykiss*. There are still few reports on the prediction of KEGG function in the gut microbiota of fish, and its effects and specific mechanisms require further study.

### 4.5. Gut immunity Gene Expression

Dietary NaB supplements may regulate the function of immune cells, including mucosal immune cells and epithelial cells, which may help the body fight off the invasion of pathogens. Interleukins, growth factors, cell-stimulating factors, and tumor necrosis factors are examples of peptide-based cellular regulatory molecules collectively referred to as cytokines [69]. Normally, immunological and inflammatory responses involve cytokines. Tumor necrosis factor (TNF), which has important biological functions, increases IL-1 and IL-8 release and promotes neutrophil adherence to endothelial cells, resulting in an inflammatory response in the body [70]. NaB can improve the defense and regulation of animals against pathogens through the control of innate and adaptive immunity and the maintenance of immunological homeostasis [71,72]. Xiao et al. found that NaB was able to reduce IL-8 expression while increasing IL-10 expression in acute pancreatitis of *Pelteobagrus fulvidraco* [73]. In this study, gene expression analysis of intestinal samples taken after *O. mykiss* was fed low fish meal diets revealed that dietary 0.20% NaB significantly affected gene expression of IL-1β, IL-8, TNF-α, nuclear factor κB (NF-κB), IKK, and IκB in the intestine of *O. mykiss*. The expression of TNF-α and IL-8 genes decreased as NaB concentration increased.

**Figure 8:**
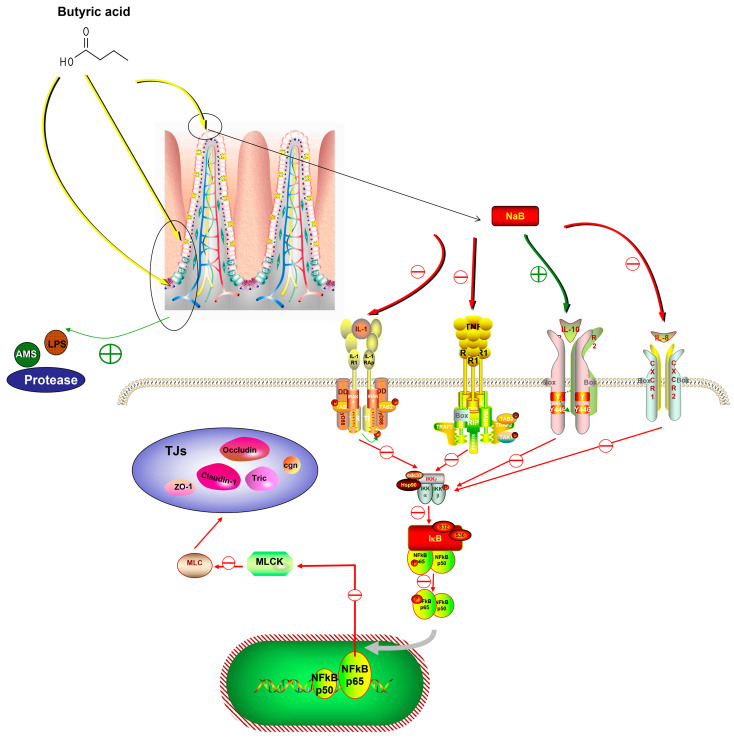
A general summary of the effect of NaB on immune function and its potential signalling pathways in the intestine of *O. mykiss*.

Unfavorable stimuli in animals first trigger the immune system, which then mildly expresses a variety of cytokines to defend the organism. However, an overactive immune response might result in inflammation. In this experiment, dietary NaB enhanced the expression of both pro-inflammatory and anti-inflammatory cytokines and regulated the expression of pathway genes associated with cytokine expression, therefore influencing the immune response in *O. mykiss* in both positive and negative ways. TNF-α and NF-κB expression were found to be reduced in turbot (*Scophthalmus maximus* L.) after 0.2% dietary NaB supplementation [29]. Infections caused by bacterial enteritis can compromise the integrity of the intestinal barrier in fish [74]. *Aeromonas hydrophila* can induce intestinal inflammation in grass carp, and it can also be disrupted by inflammatory processes caused by the excessive substitution of fish meal and fish oil in the feed [75]. Enteritis can also be caused by damage to the intestinal mucosa due to frequent consumption of a diet high in soybean meal [76]. Therefore, dietary NaB may play an anti-inflammatory role in reducing intestinal inflammation by inhibiting the NF-κB-P65 inflammatory signaling pathway, thus protecting the intestinal mucosa (Figure 8) [77,78].

Animals with healthy intestines have a variety of bacteria, some of which produce endotoxins that cause harmful reactions and trigger the production of intestinal inflammatory mediators and cytokines, leading to a disruption of the intestinal barrier [79,80]. An increase in intestinal mucosal and vascular permeability, which is an indication of intestinal barrier damage, increases the risk of pathogens and endotoxins moving from the intestine to local lymph nodes, portal veins, and the peripheral blood system. This can cause pancreatic necrosis and organ failure in *O. mykiss*. Tight junctions (TJ), which prevent bacteria, lipopolysaccharides, and other potentially dangerous substances from entering the bloodstream from the intestinal lumen, make up a significant part of the intestinal barrier in the fish gut [81]. Cldn-1, Ocln, and ZO-1 are the three primary TJ proteins, and they improve the intestinal barrier by reducing paracellular permeability [82]. TNF-α, which increases TJ permeability by controlling the expression of the MLCK gene in Caco-2 cells through the NF-κB signaling pathway, has also been linked to intestinal barrier failure [83]. In this study, dietary NaB may protect intestinal barrier function by increasing the expression of TJ-related proteins, Ocln, and ZO-1 genes. Some scientists propose that intestinal epithelial NF-κB regulates MLCK activity and directly targets MLCK. Specifically, activated NF-κB releases p65, which translocates to the cytosol of the nucleus and binds to the MLCK promoter to increase MLCK. MLCK-mediated MLC phosphorylation subsequently causes myosin contraction and TJ dysregulation. NaB prevents the master inflammatory regulator NF-κB and also prevents MLCK-mediated barrier erosion (Figure 8). Therefore, this study showed that dietary NaB may protect the integrity of the intestinal epithelium in *O. mykiss* by preventing TJ dysfunction and barrier degradation caused by MLCK.

### 4.6. Survival upon the A. salmonicida Challenge of Triploid O. mykiss

*A. salmonicida* is a short, Gram-negative bacterium of the genus *Aeromonas* that causes boils or ulcers in salmon and trout. Typical symptoms of the disease include the formation of characteristic abscesses on the side or tail of the fish; in severe cases, the abscesses ulcerate, ulcers form, and the liver and adipose tissue bleed on autopsy [84]. It has been found that the virulence of *A. salmonicida* is associated with various virulence factors, including the A-layer protein, glycerophospholipid cholesterol acyltransferase (GCAT), proteases, and siderophores. GCAT, in particular, has been found to significantly influence the pathogenicity of *A. salmonicida*, exhibiting cytotoxicity, hemolytic activity, and thermal stability [85]. According to Scott et al., salmon with scabies exhibit an enlarged spleen, subhepatic perithelial hemorrhage, pitting hemorrhage in parenchymal tissues, anorexia or oligophagia, and a stomach and intestine filled with blood and mucus, all of which are connected to the hemolytic and cytotoxic properties of *A. salmonicida* [86]. In this study, the group fed the 0.20% NaB diet and challenged with *A. salmonicida* had a significantly lower cumulative mortality compared to group G1 (*p* < 0.05). Piazzon et al. [61] obtained similar results in the teleostean gilthead sea bream (*Sparus aurata*). Butyric acid, a direct source of energy for intestinal cells, promotes the proliferation of intestinal epithelial cells, restores the ANF-damaged intestinal barrier, reduces colitis, and may be responsible for the decrease in cumulative mortality. NaB also significantly increased the mRNA expression of TJ proteins and protected the intestinal barrier in *O. mykiss* [87]. The increased colonization of probiotic bacteria in the intestine following NaB administration may also contribute to the resistance of *O. mykiss* to *A. salmonicida* infection. Furthermore, more research has to be done to determine the precise mechanism by which NaB protects against bacterial infection.

## 5. Conclusions

Dietary 0.20%–0.22% NaB levels improved the WGR and SGR of juvenile *O. mykiss* fed a 15% fish meal diet and reduced the severity of ANF-induced enteropathy by enhancing digestion enzymes and improving intestinal morphology. Dietary NaB increased mRNA expression of TJ proteins (Ocln, Cldn-3, and ZO-1) while decreasing mRNA expression of pro-inflammatory cytokines (IL-1β, IL-8, and TNF-α), NF-κB, and MLCK-MLC, suggesting that it protects against pathogenic bacterial infections by strengthening the intestinal mucosal barrier. In addition, the gut microbial composition was more favorable for *O. mykiss* health with the inclusion of 0.20% NaB in the diet. Furthermore, when challenged with *A. salmonicida*, dietary 0.20% NaB increased the survival rate of *O. mykiss*.

## Figures and Tables

**Table 1:** Ingredients and their contents of the test diets (air-dry basis, %).

Ingredients	Groups				
	G1 (0.00%)	G2 (0.10%)	G3 (0.20%)	G4 (0.30%)	G5 (0.40%)
Soybean oil ^1^	5.38	5.38	5.38	5.38	5.38
Fish meal ^2^	15.00	15.00	15.00	15.00	15.00
Wheat flour ^1^	24.40	24.40	24.40	24.40	24.40
Fish oil ^2^	5.00	5.00	5.00	5.00	5.00
Compound amino acids ^3^	17.02	17.02	17.02	17.02	17.02
Soybean meal ^1^	21.60	21.60	21.60	21.60	21.60
Beer yeast ^1^	6.00	6.00	6.00	6.00	6.00
Premix ^4^	4.00	4.00	4.00	4.00	4.00
Ca(H_2_PO_4_)_2_^5^	1.00	1.00	1.00	1.00	1.00
Calcium propionate ^5^	0.20	0.20	0.20	0.20	0.20
Microcrystalline cellulose ^6^	0.40	0.30	0.20	0.10	0.00
Sodium butyrate (98.50%) ^7^	0.00	0.10	0.20	0.30	0.40
Nutrient proximate levels ^8^					
Crude protein	37.63	35.11	35.66	37.61	35.66
Crude lipid	15.95	15.56	15.61	15.37	15.56
Ash	3.71	3.73	3.72	3.70	3.77
Moisture	8.33	8.17	8.39	8.53	8.25
Gross energy (MJ/kg) ^9^	21.55	21.03	21.29	21.12	21.83

^1^ Dalong Feed Company, Harbin, China. ^2^ HaiYuan Biotech Company, Guangxi, China. ^3^ Amino acid content: Lys 2.13%; Arg 0.62%; His 0.46%; Val 1.72%; Lle 1.46%; Pro 0.86%; Phe 0.90%; Asp 1.78%; Tyr 0.82%; Ala 1.42%; Cys 0.15%; Glu 3.63%; Leu 2.01%; Gly 0.72%; Met 0.66%. ^4^ Composition of the premix provided (4%): VE 60 IU, VD_3_ 3 000 IU, VC 0.10 g, VB_2_ 30 mg, VB_6_ 15 mg, VB_1_ 15 mg, VK_3_ 5 mg, VA 4.50 mg, VB_12_ 500 μg, MgSO_4_·7H_2_O 2.00 g, KCl 1.50 g, NaCl 0.50 g, nicotinic acid 0.175 g, ZnSO_4_·7H_2_O 0.15 g, inositol 0.10 g, FeSO_4_·7H_2_O 0.10 g, MnSO_4_·4H_2_O 0.10 g, calcium pantothenate 0.05 g, CuSO_4_·5H_2_O 0.02 g, folic acid 5 mg, CoCl_2_ 5 mg, KI 3 mg, Na_2_SeO_3_ 3 mg, biotin 2.5 mg. ^5^ TianDa Chemical Reagent Co., Tianjin, China. ^6^ Beijing Labgic Technology Co., Beijing, China. ^7^ Sigma-Aldrich, St. Louis, MO, USA. ^8^ Nutrient levels were determined values. ^9^ Calorimeter (IKA C-200, Staufen, Germany).

**Table 2:** PCR amplification procedure and real-time PCR primer sequences.

Items	PCR Reaction Solution Preparation			PCR Amplification Procedure	
	Reagent	Consumption	Concentration	Procedure	Instrument
RT-PCR	TB Green Premix Ex Taq II (Tli RNaseH Plus) ^1^	10 μL	2×	Step 1: Reps: 1 95 °C 30 s Step 2: Reps: 40 95 °C 5 s 60 °C 34 s	7500 Real-Time PCR System; Applied Biosystems, Waltham, MA, USA
	ROX Reference Dye II ^1^	0.4 μL	50×		
	PCR Forward Primer ^2^	0.8 μL	10 μM		
	PCR Reverse Primer ^2^	0.8 μL	10 μM		
	cDNA ^3^	2 μL	50 ng/μL		
	DEPC H_2_O ^4^	6 μL			
PCR	FastPfu Buffer ^5^	4 μL	5×	Step 1: Reps: 1 95 °C 3 min Step 2 Reps: 27 95 °C 30 s 55 °C 45 s 72 °C 45 s Step 3 72 °C 10 min	Gene Amp 9700; Applied, USA
	dNTPs ^5^	2 μL	2.5 mM		
	FastPfu Polymerase ^5^	0.4 μL			
	Primer 338F (5′-ACTCCTACGGGAGGCAGCAG-3′) ^2^	0.8 μL	5 μM		
	Primer 806R (5′-GGACTACHVGGGTWTCTAAT-3′) ^2^	0.8 μL	5 μM		
	Template DNA ^6^	10 ng			

^1^ RR820A; TaKaRa, Beijing, China. ^2^ Comate Bioscience Company Limited, Changchun, China. ^3^ TRIzol Reagent; Ambion, USA. ^4^ NR0001; Leagene Biotechnology, Beijing, China. ^5^ AP221-02; TransGen, Beijing, China. ^6^ E.Z.N.A. Soil DNA Kit, Norcross, GA, USA; Omega Bio-tek, Norcross, GA, U.S.

**Table 3:** Primer sequences.

Genes	Primer Sequences Forward and Reverse (5’—3’)	Accession Number	Annealing Temp (°C)	Primer Efficiency %
β-actin	F: GGACTTTGAGCAGGAGATGG R: ATGATGGAGTTGTAGGTGGTCT	XM_044093545.1	61.40	99.65
TGF-β	F: ACTGTGCCCCTGCAAGTCT R: CTGTGCTGTCCTACGCTCTG	X99303	55.90	96.00
TNF-α	F: GGGGACAAACTGTGGACTGA R: GAAGTTCTTGCCCTGCTCTG	AJ278085.1	58.40	93.00
IKK	F: CTGCATCGCTACCTCAGGAG R: TAAGAAAACACCCCTGGGCC	BT073400.	60.00	96.99
IκB-α	F: GGCAGAATTGAAGTGGTCGC R: GCTTCTGGGACCTGGAGTTC	BT074224.1	60.00	94.00
MLCK	F: GTGTGTGTGCCGGAAAGTTC R: ATCATAGGCCCCCAGACACT	NC_048576.1	60.00	95.00
MLC	F: GCCCGTTTCCTGTGCAATTT R: GCTTGGGTCGCTAAT	XM_021565852.2	60.00	99.44
IL-8	F: GAATGTCAGCAGCCTTGTC R: TCCAGACAAAYCYCCYGACCG	AJ310565.1	60.30	98.00
ZO-1	F: AAGGAAGGTCTGGAGGAAGG R: CAGCTTGCCGTTGTAGAGG	XM_036980662.1	60.00	98.00
tric	F: GTCACATCCCCAAACCAGTC R: GTCCAGCTCGTCAAACTTCC	KC603902	60.00	96.00
IL-1β	F: ACCAGCCTTGTCGTTGTG R: GTTCTTCCACAGCACTCTCC	AB010701.1	57.10	96.00
Ocln	F: CAGCCCAGTTCCTCCAGTAG R: GCTCATCCAGCTCTCTGTCC	GQ476574	58.00	96.43
NF-κB	F: CAGGACCGCAACATACTGGA R: GCTGCTTCCTCTGTTGTTCCA	XM_031794907.1	58.40	96.00
Cgn	F: CTGGAGGAGAGGCTACACAG R: CTTCACACGCAGGGACAG	BK008767	56.00	98.00
IL-10	F: CGACTTTAAATCTCCCATCGAC R: GCATTGGACGATCTCTTTCTTC	AB118099.1	65.00	94.00
cldn-3	F: TGGATCATTGCCATCGTGTC R: GCCTCGTCCTCAATACAGTTGG	BK007964	60.00	93.00

Abbreviations: IL, Interleukin; TGF-β, Transforming growth factor-β; TNF-α, Tumor necrosis factor-α; IκB-α, inhibitor of κB α; IKK, IκB kinase; NF-κB, Nuclear factor-kappa B; MLCK, myosin light chain kinase; MLC, myosin light chainmyosin; ZO-1, Zonula occluden-1; tric, tricellulin; Ocln, occluding; Cgn, Cingulin; cldn-3, claudin-3.

**Table 4:** Growth performance, feed conversion of triploid *O. mykiss* in five groups during the experiment.

Items	Groups				
	G1	G2	G3	G4	G5
Initial body weight (g)	9.05 ± 0.04	8.67 ± 0.12	8.64 ± 0.11	8.87 ± 0.03	8.89 ± 0.07
Final body weight (g)	26.09 ± 0.18 ^ab^	25.29 ± 0.25 ^a^	26.27 ± 0.2 ^b^	25.86 ± 0.35 ^ab^	26.18 ± 0.34 ^ab^
WGR (%)	188.29 ± 2.01 ^a^	191.69 ± 2.2 ^ab^	204.01 ± 4.81 ^b^	191.49 ± 3.13 ^ab^	194.65 ± 6.08 ^ab^
Survival rate (%)	98.89 ± 1.11	100.00 ± 0.00	97.78 ± 1.11	98.89 ± 1.11	97.78 ± 2.22
FCR	1.29 ± 0.01 ^ab^	1.32 ± 0.01 ^a^	1.24 ± 0.02 ^b^	1.30 ± 0.02 ^ab^	1.27 ± 0.03 ^ab^
SGR (%/d)	1.89 ± 0.01 ^a^	1.91 ± 0.01 ^ab^	1.99 ± 0.03 ^b^	1.93 ± 0.04 ^ab^	1.91 ± 0.02 ^ab^
PER	1.94 ± 0.02 ^ab^	1.89 ± 0.02 ^a^	2.01 ± 0.03 ^b^	1.93 ± 0.04 ^ab^	1.97 ± 0.05 ^ab^
CF	1.17 ± 0.02	1.24 ± 0.03	1.17 ± 0.02	1.23 ± 0.02	1.17 ± 0.02
HSI (%)	4.28 ± 0.21 ^b^	3.75 ± 0.24 ^ab^	3.45 ± 0.25 ^ab^	3.06 ± 0.21 ^a^	3.46 ± 0.19 ^ab^
VSI (%)	19.64 ± 0.57 ^b^	17.48 ± 0.68 ^a^	17.81 ± 0.58 ^ab^	17.78 ± 0.43 ^ab^	18.09 ± 0.74 ^ab^

Note: Values are presented as mean ± S.E. (*n* = 3). Values in the same column with different superscript letters are significantly different (*p* < 0.05). Abbreviations: WGR, weight gain rate; SGR, specific growth rate; FCR, feed conversion; PER, protein efficiency ratio; CF, condition factor; HSI, hepatosomatic index; VSI, viscerosomatic index.

**Table 5:** Body composition of triploid *O. mykiss* in five groups (wet weight, %).

Indices	Groups				
	G1	G2	G3	G4	G5
Crude protein	14.45 ± 0.15	13.93 ± 0.12	14.07 ± 0.43	14.83 ± 0.63	14.45 ± 0.15
Crude lipid	9.78 ± 0.32	10.02 ± 0.07	9.4 ± 0.04	9.68 ± 0.37	9.13 ± 0.03
Ash	2.38 ± 0.07	2.23 ± 0.03	2.47 ± 0.05	2.43 ± 0.13	2.42 ± 0.03
Moisture	73.04 ± 0.17	72.87 ± 0.29	73.11 ± 0.24	72.61 ± 0.38	73.47 ± 0.32

Note: Values are presented as mean ± S.E. (*n* = 3).

**Table 6:** The intestinal digestive enzyme and intestinal morphology in triploid *O. mykiss* in five groups.

Indices	Groups				
	G1	G2	G3	G4	G5
Intestinal digestive enzyme					
LPS (U/g prot)	13.28 ± 0.07 ^ab^	13.80 ± 0.78 ^ab^	19.96 ± 0.43 ^c^	15.10 ± 0.54 ^b^	12.40 ± 0.33 ^a^
Trypsin (U/mg prot)	2868.24 ± 192.79 ^b^	4332.58 ± 141.72 ^c^	5553.76 ± 61.09 ^d^	2364.32 ± 262.44 ^b^	1580.33 ± 49.06 ^a^
AMS (U/mg prot)	0.11 ± 0.01 ^ab^	0.12 ± 0.02 ^ab^	0.18 ± 0.03 ^b^	0.11 ± 0.00 ^ab^	0.07 ± 0.01 ^a^
Intestinal morphology (μm)					
Villus length	375.1 ± 9.72 ^a^	774.31 ± 17.99 ^bc^	838.48 ± 61.58 ^c^	641.68 ± 30.86 ^b^	313.18 ± 19.46 ^a^
Villus width	149.86 ± 4.15 ^b^	203.76 ± 15.24 ^c^	256.99 ± 8.9 ^d^	156.92 ± 9.62 ^b^	116.69 ± 15.31 ^a^
Muscular layer Thickness	58.99 ± 6.35 ^a^	80.27 ± 1.80 ^bc^	83.58 ± 3.86 ^c^	69.08 ± 3.45 ^ab^	66.79 ± 2.15 ^a^

Note: Values are presented as mean ± S.E. (*n* = 3). Values in the same column with different superscript letters are significantly different (*p* < 0.05). Abbreviations: LPS, lipase; AMS, amylase.

## Data Availability

The original contributions presented in the study are included in the article, and further inquiries can be directed to the corresponding authors.
